# Isolated Case of Bioterrorism-related Inhalational Anthrax, New York City, 2001

**DOI:** 10.3201/eid0906.020668

**Published:** 2003-06

**Authors:** Timothy H. Holtz, Joel Ackelsberg, Jacob L. Kool, Richard Rosselli, Anthony Marfin, Thomas Matte, Sara T. Beatrice, Michael B. Heller, Dan Hewett, Linda Moskin, Michel L. Bunning, Marcelle Layton, the New York City Anthrax Investigation Working Group

**Affiliations:** * Centers for Disease Control and Prevention, Atlanta, Georgia, USA; † New York City Department of Health, New York, New York, USA; ‡ Centers for Disease Control and Prevention, Fort Collins, Colorado, USA; § New York Academy of Medicine, New York, New York, USA; ¶ Office of the Surgeon General, Bolling Air Force Base, Washington, D.C., USA

**Keywords:** *B. anthracis*, inhalational anthrax, bioterrorism, research

## Abstract

On October 31, 2001, in New York City, a 61-year-old female hospital employee who had acquired inhalational anthrax died after a 6-day illness. To determine sources of exposure and identify additional persons at risk, the New York City Department of Health, Centers for Disease Control and Prevention, and law enforcement authorities conducted an extensive investigation, which included interviewing contacts, examining personal effects, summarizing patient’s use of mass transit, conducting active case finding and surveillance near her residence and at her workplace, and collecting samples from co-workers and the environment. We cultured all specimens for *Bacillus anthracis*. We found no additional cases of cutaneous or inhalational anthrax. The route of exposure remains unknown. All environmental samples were negative for *B. anthracis*. This first case of inhalational anthrax during the 2001 outbreak with no apparent direct link to contaminated mail emphasizes the need for close coordination between public health and law enforcement agencies during bioterrorism-related investigations.

After the World Trade Center attack on September 11, 2001, the possibility of bioterrorism in New York City (NYC) became a preeminent concern at the Department of Health (DOH). Active syndromic surveillance by emergency department for bioterrorism-related illnesses was initiated in 15 hospitals, and frequent broadcast alerts were sent by email and fax to all NYC emergency departments, commercial and hospital laboratories, infection-control programs, and selected providers (*1*).

After the announcement of the inhalational anthrax index case in Florida on October 4 and the cutaneous anthrax index case in NYC on October 12, DOH enhanced its active surveillance activities citywide (*2*). Detailed diagnostic and treatment protocols were provided through a broadcast alert system and the DOH website to the medical and laboratory community, including emergency departments, intensive-care units, infectious disease and infection-control specialists, dermatologists, and laboratories. A provider hotline was established for rapid referral and evaluation of suspect cases. Broadcast fax alerts also were sent to veterinarians to request reporting of suspect animal cases. In addition, the emergency department–based syndromic surveillance system was expanded to 29 hospitals to augment DOH’s ability to detect a large, covert bioterrorist event. The medical examiner’s office was asked to notify DOH of any suspicious deaths from unexplained sepsis or respiratory causes.

During October, four simultaneous investigations were conducted at news media outlets where cutaneous anthrax cases were detected among employees (M. Phillips, et al., unpub. data). All interviews were performed by teams of investigators from DOH, Centers for Disease Control and Prevention (CDC), and law enforcement on the basis of pre-established agreements between DOH and the New York field office of the Federal Bureau of Investigation (FBI), and its associated Joint Terrorism Task Force (a task force between the NYC Police Department and FBI). By the end of October 2001, seven laboratory-confirmed or suspected cutaneous anthrax cases had been reported in NYC. All case-patients were thought to have been exposed through direct contact with contaminated mail addressed to media outlets and postmarked on September 18 (*3*). The last known contaminated letters were postmarked on October 9 from Trenton, New Jersey, to Senators Thomas Daschle and Patrick Leahy in Washington, D.C.

## Case Confirmation

On October 28, 2001, a local hospital reported a suspected case of inhalational anthrax to DOH. The case-patient was a 61-year-old female with a 3-day history of progressive weakness, chest heaviness, myalgia, cough, and shortness of breath. She was admitted to intensive care with respiratory failure, emergently intubated before being interviewed, and treated with multiple antibiotics and diuretics for a presumptive diagnosis of community-acquired pneumonia, congestive heart failure, or inhalational anthrax (*4*). On October 29, nonmotile, gram-positive rods in long chains were isolated from routine blood cultures, and her antibiotic therapy was adjusted to provide for enhanced coverage of inhalational anthrax. That evening, *Bacillus anthracis* was preliminarily identified from her blood culture isolate and from pleural, fluid, and bronchial washings by polymerase chain reaction (PCR) at the DOH Public Health Laboratory and CDC. The following day, pleural and blood isolates were confirmed as *B. anthracis* by gamma phage lysis and direct fluorescent antibody testing. The case-patient died on October 31. *B. anthracis* isolates were subtyped at CDC by multiple-locus variable-number tandem repeat analysis (MLVA) and sequencing of the *pag*A gene. All isolates were MLVA genotype 62 and *pag*A genotype I, the same genotype as all other isolates from the 2001 anthrax outbreak in Florida, New Jersey, Washington, D.C., and Connecticut (*5*).

We report the results of the epidemiologic and environmental investigation by DOH, CDC, and local and federal law enforcement agencies in response to this isolated case of inhalational anthrax. The objectives of our investigation were to determine the time, location, and route of exposure; to identify any additional cases of cutaneous or inhalational anthrax; to determine whether this case was an isolated case or sentinel case of a larger outbreak; and to guide our public health response.

## Methods

### Case Investigation

Immediately after confirming the case-patient’s diagnosis, epidemiologists from DOH and CDC and a detective and special agents from the Joint Terrorism Task Force formed joint investigative teams to ensure the rapid and efficient sharing of relevant information between the epidemiologic and criminal investigations. To identify the time and location where the case-patient might have been exposed to anthrax during the 60 days before illness onset, detectives from the NYC Police Department and FBI along with local and federal epidemiologists performed joint interviews of the patient’s social, work, and neighborhood contacts. We chose a 60-day period on the basis of the range of the inhalational anthrax incubation period during the Sverdlovsk anthrax outbreak in 1979 (*6*). We conducted regular interagency meetings to analyze new information collaboratively and strategize about the next steps of the investigations.

We collected information about the case-patient’s habits and activities through interviews with co-workers, neighbors, acquaintances, and a mail carrier to uncover any potentially relevant personal details, including places she frequented, and her social contacts. Investigators searched the case-patient’s apartment, examined personal effects, reviewed telephone and financial records, and visited four post offices that she was known or thought to have used. To locate persons who might have information regarding activities during the incubation period, we displayed the case-patient’s photograph in churches that she reportedly attended and in Chinatown, which she frequented. Employees from 15 businesses near the case-patient’s apartment complex and work were interviewed. Members of the NYC Anthrax Investigation Team also met with investigators of the other unexplained inhalational case in Connecticut (*7*).

Using the identification number from a subway transit card issued to the case-patient by the New York City Metropolitan Transportation Authority (MTA), we obtained data about her transit activity from October 22 to October 26, indicating which buses and subway stations she entered in the week before onset of illness. The MTA also identified a previous transit card number with boarding times that matched the pattern of subway use linked to her card. Using this information, we were able to approximate her bus and subway travel during the previous month (September 21–October 20).

### Case Finding

Our investigation included active case finding and surveillance for additional anthrax cases at the case-patient’s workplace and in her apartment complex. We asked all co-workers, patients, and visitors who had spent >1 hour at her workplace during the preceding 2 weeks to report for an interview; at the interview, they were offered antibiotic prophylaxis. We also used the following information to identify additional suspect cases: 1) the hospital’s employee health department list of all employees who had been evaluated for fever during the previous 2 weeks; 2) the human resources department list of employees who had missed >1 day of work during this period; 3) interviews regarding symptoms in occupants from 27 of 28 of the neighboring apartments in her complex; and 4) a community meeting in the case-patient’s Bronx neighborhood in which the case was discussed and neighbors were encouraged to report illnesses or skin lesions suggestive of anthrax.

In addition, active surveillance for suspect cases was established with the U.S. Postal Service and MTA in NYC because of concern regarding potential exposures in post offices, from mail, or in the subway system. Any ill employee was contacted by DOH staff by telephone and asked about symptoms. Any suspect cases were referred for immediate evaluation and follow-up.

### Environmental and Laboratory Investigation

To search for evidence of a recent exposure to aerosolized anthrax-containing particles, we collected both nasal swabs and environmental samples. On October 29, we collected nasal swabs from 28 co-workers who worked near the case-patient. We used four sampling techniques for environmental surface samples: dry Bacti-swab (Remel Inc., Lenexa, KS) sampling, wet swabs, composite dry swabs, and HEPA vacuum samples (*8*).

From October 29 through December 12, sampling was performed in and around the case-patient’s apartment and from selected personal effects: from the hospital where she worked, with an emphasis on the mail bins, mailroom, and stockrooms she frequented; in an acquaintance’s apartment where she had slept during the incubation period; from a neighborhood post office where she had purchased a postal money order 2 days before illness onset; from three mail-processing facilities and two post offices that served her home and workplace; and from two businesses near her apartment complex (Table). Subway stations were selected for sampling on the basis of the pattern of her subway use gleaned from her MTA transit card (Figure). Nasal swabs, bulk specimens, dry surface swabs, composite swabs, and vacuum sock samples were analyzed by the DOH Public Health Laboratory and the U.S. Department of Defense by using standard culture techniques and PCR analysis (*9*).

**Table:** Summary of environmental test sampling in inhalational anthrax investigation, New York City, 2001^a^

Date in 2001	Location	Items	Wet swabs	Dry swabs	Vacuum filters	
10/29	Case-patient’s hospital workplace: old and new mail room	Mail cubby spaces, air intake, lights, desk top, computer keyboard, and air-conditioning air-intake filters		10		
	Case-patient’s workplace: workroom, basement rooms, and elevator	Desk tops, floor, lighting and vents, door casings, walls, and ceilings		24		
	Admitting hospital	Case-patient’s clothing and personal property from admission		7		
	Case-patient’s workplace	Nasal swabs of hospital coworkers		28		
10/30	Case-patient’s apartment	Appliance tops, mail bins, table tops, post office receipts, window sills, light fixtures, trash cans, and bank cards		40		
10/31	Case-patient’s workplace locker and workspace	Locker contents (clothing, shoes, lab coat, personal belongings), elevator exhaust blades and cages, air-intake grills, door jambs, and computer cooling fans		52	4	
11/1	Manhattan post office A	Computer fan intakes, light fixtures, teller computer monitors, air-intake grills, counter surfaces, and vacuum sample of multiple mail-sorting cubbies		12	1	
	Case-patient’s apartment	Mailbox, adjacent mailboxes, elevator fans, refrigerator, baseboards, personal items, clothes hampers, television screen, boxes containing recent mail, light fixtures, vacuum cleaner dust, and cooling fans		29		
	Case-patient’s apartment	Closet, hanging garments, bedroom clothes, and hallway bureau			8	
	Personal items from case-patient’s apartment	Slippers, shoes, hats, pieces of recent mail, address book, pictures, and stuffed animal		9		
	Office of the Chief Medical Examiner	Autopsy room, sink, table, cutting floor, and floor		31		
11/2	Bronx post office A	Multiple mail slots, sorting areas, and intake grills of ventilation systems		15	3	
	Manhattan post office B	Mail-sorting areas and ventilation system, fluorescent light fixtures		9		
11/3	Case-patient’s workplace (basement storage)	Door jams, light fixtures, conduits, air-conditioner intake filters, pipes		8		
11/4	Bronx mail sorting and distribution center	Optical character readers, air intake of ventilation system, manual sorting stations, dead-letter repository, digital bar code sorters (including the machine that sorts mail to case-patient’s home), overhead air filters		25		
	Bronx parcel post office B	Sorting stations, registered mail cage and delivery trucks		8		
	Case-patient’s apartment	Powder samples		9		
11/5	Bronx post office box A	Case-patient’s post office box		1		
	Friend’s home	Dining room and bedroom surfaces		2		
	Case-patient’s workplace (basement storage)	Storage lockers, urinal, plumbing, switches, and bulbs		7		
	Case-patient’s workplace work station	Fan, light, and pipe		5		
11/6	Case-patient’s personal effects from apartment	Unspecified substance in case-patient’s wallet		1		
11/11	Subway line, #6 Lexington Ave line (including Grand Central Station) and 1 N/R station used by case-patient	Multiple dust-collecting areas along platform and multiple air-intake filters of recently installed air-conditioner system in Grand Central Station	120		19	
	Four subway stations not known to be used by case-patient (control stations)	Multiple dust-collecting areas along platform	76			
11/30	Two businesses near to case-patient’s apartment that received mail from Trenton postmarked October 9	Mail bins, scissors, letter openers, desk, phone, window, computer, air-conditioner, and copier		40		
12/12	Case-patient’s personal effects from apartment associated with hypothesized exposure locations or pathways	Coat, shoes, tennis shoes, slippers, hats, scarf and lab coat, hospital identification card, documents dated in mid-October including mail, money orders, bank withdrawal slips, receipts, phone, and perfume bottles			18	
Total			196	372	53	

^a^Left column indicates the date samples were taken. Aggressive sampling was performed by intensively and individually vacuuming each item by using a HEPA filter vacuum sock. Collected material was processed through a membrane to selectively remove extraneous material (e.g. hairs, which can be saved for later forensic examination) and concentrate spores in the filtrate. Filtrate was then analyzed by polymerase chain reaction and cultured for *Bacilllus anthracis*. Material in perfume bottles was cultured directly. Tests on samples were completed on 12/12/2001.

**Figure:**
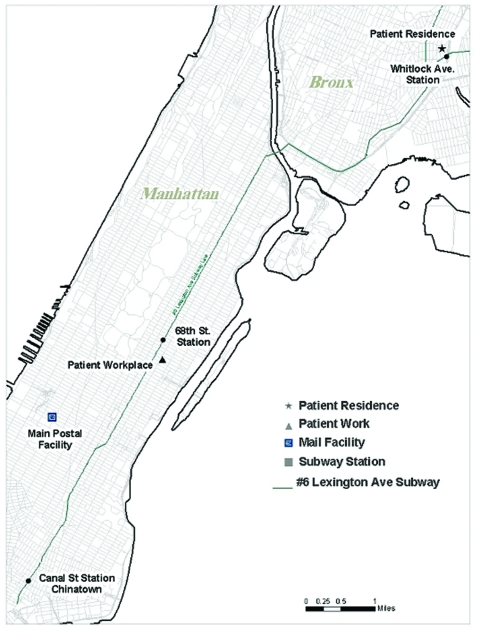
Key Manhattan and Bronx locations during investigation of inhalational anthrax, New York City, 2001.

### Mail Investigation

We obtained data from the U.S. Postal Service on the final destinations for first-class mail sorted from October 9 to October 16 on the same digital bar code–sorting machine in the Trenton, N.J., processing and distribution center that sorted the letters addressed to Senators Daschle and Leahy. Bulk mail is presorted and not labeled with codes. We focused on first-class mail that was then delivered to the same zip codes as our case-patient’s home and work addresses.

### Public Health and Prevention Activities

On October 30, in conjunction with the hospital, DOH established an antibiotic prophylaxis clinic for persons who were considered at potential risk for inhalational anthrax; at this time, we expanded the initial prophylaxis given to include the 28 staff persons who worked in the hospital basement near the stockroom and mailroom (*10*). All co-workers, patients, and visitors who had spent >1 hour at the case-patient’s workplace in the preceding 2 weeks were recommended for antibiotic prophylaxis evaluation. Initially, persons were offered 10 days of doxycycline or ciprofloxacin (*11*), pending results from the epidemiologic and environmental investigations to determine whether the hospital was the site of exposure. We offered amoxicillin to children on the basis of the susceptibility pattern of the other *B. anthracis* isolates associated with this outbreak (following CDC guidelines) (*12*).

## Results

### Case Investigation

The case-patient was a resident of an apartment in a predominately Hispanic section of the Bronx; she lived alone. She had no known family members in NYC and a limited number of close acquaintances. A refugee from Vietnam, she had lived in the United States for 26 years. Her acquaintances at work and in her neighborhood reported her daily routine and usual activities to be habitual, with a regular work schedule, visits to Chinatown in Manhattan to shop, and trips to post offices and department stores near her workplace and home. Although reportedly friendly and generous, she lived a solitary life. Her apartment was clean and tidy. We collected no information suggesting that she had traveled outside NYC during the 60-day period before onset of illness. Neighbors did not recall any recent visitors to her home. From information received from MTA, we ascertained that she rode the No. 6 Lexington Avenue subway almost daily, traveling from her home in the Bronx to her workplace in Manhattan (Figure).

She had worked full time in an East Side Manhattan hospital for 12 years, delivering supplies from basement stockrooms to clinics and wards within the hospital. She had not missed any work in the several weeks before onset of illness. She did not directly work with or sort mail. The hospital’s mail was sorted in a section of one of these stockrooms, where it was placed into wooden mail slots. The mailroom and stockroom staff reported no suspicious packages or letters.

We constructed a timeline of her activities in the several weeks preceding her illness. Although her subway transit card, work records, financial records, and sales receipts allowed us to account for some of her activities, approximately 40% of the nonwork-related hours before her death remained unaccounted for. The criminal investigation found no suspicious letters or activity to connect the case-patient to any of the known anthrax-laden postmarked letters, and no evidence existed that she had visited any of the media sites in NYC affected by this outbreak. Shared information with the investigators in Connecticut indicated that she and the case-patient in that state had little in common.

### Case Finding

A total of 232 coworkers, occupants from 27 of 28 neighboring apartment units, 35 acquaintances, and 1,675 hospital patients and visitors were screened for symptoms of cutaneous or inhalational anthrax in connection with this single case. A total of 69 persons with respiratory symptoms and 21 with suspicious skin lesions were followed up by medical evaluation or telephone call. None of these persons were diagnosed with inhalational or cutaneous anthrax.

We contacted all hospital employees listed as ill on absentee (n=60) and employee health (n=88) lists and found them to have recovered from minor illnesses or injuries. Enhanced surveillance in NYC hospitals, the U.S. Postal Service, and emergency departments in NYC have continued since 2001 to 2003. No further cases of anthrax have been identified in NYC.

### Environmental Investigation

All 621 environmental samples tested negative for *B. anthracis* by culture and PCR: workplace (n=138), apartment (n=86), personal effects (n=35), an acquaintance’s apartment (n=2), businesses near her apartment (n=40), post offices (n=74), the Office of the Chief Medical Examiner (n=31), and subway (n=215) (Table).

### Mail Investigation

The U.S. Postal Service identified letters delivered to the case-patient’s work address, but not to her home address, that were postmarked on October 9 in Trenton and sorted by the same digital bar code–sorting machine as the letters sent to Senators Daschle and Leahy. However, all other hospitals with the same zip code as her workplace and many other places in the city also received mail postmarked on October 9 by this same machine. The closest location to her home where an October 9–postmarked letter from the Trenton facility was delivered was a commercial property two blocks from her apartment complex. No letters postmarked October 9 were identified at the two businesses located at this property, no persons at either business had been ill with symptoms suggestive of anthrax infection, and all environmental samples were negative.

Five digital bar code–sorting machines at the main Manhattan postal distribution center tested positive for *B. anthracis* in late October during the initial investigation of the media-related cutaneous cases. None of these five contaminated machines routinely performed final sorting of mail for the 5-digit zip codes that included the case-patient’s home and workplace. The sorting machines that routinely sorted mail sent to the case-patient’s workplace and home zip codes tested negative.

### Public Health Intervention

On the recommendation of DOH, the local hospital where the case-patient worked closed voluntarily for 10 days after her diagnosis. DOH and local hospital screened and offered prophylaxis for anthrax to 232 coworkers and 1,675 hospital patients and visitors beginning on October 29. After all of the initial environmental samples at the case-patient’s workplace tested negative and no additional suspect cases were identified, DOH recommended that all persons discontinue antibiotic prophylaxis on November 7.

## Discussion

This investigation focused on the first and only case of inhalational anthrax in NYC during the 2001 anthrax outbreak. This case was the first of two that occurred during this outbreak that did not have an apparent direct link to contaminated mail (*13*). No other confirmed cutaneous or inhalational anthrax infections associated with this case were identified during the 2001 outbreak. The timing, location, and route of the case-patient’s exposure remain unknown. Our epidemiologic and environmental investigations yielded no firm indications regarding when, where, how, or why this case-patient was infected. Despite concern that she might represent the sentinel case of a much larger outbreak, subsequent surveillance showed that this case was probably an isolated case of inhalational anthrax and not part of a larger local outbreak.

During this investigation, we considered multiple hypothetical scenarios to explain how the case-patient could have been exposed to *B. anthracis*. Natural exposure was thought to be unlikely because the last case of naturally acquired anthrax in NYC occurred in 1947, the case-patient had no known risk factors for natural infection, and her isolate was genotypically indistinguishable from other isolates from the 2001 anthrax outbreak. Although exposure to an intentionally contaminated letter was considered the most likely hypothesis from the onset, our case-patient had none of the risk factors for anthrax infection identified among earlier cases (e.g., working for the news media or government, handling mail) (*14*). Other less likely explanations for her death—that she was associated with the terrorists or targeted purposely, that she was present when a small-scale attack intentionally occurred, or that she happened to pass by when a small amount of anthrax spores were accidentally released by the perpetrator—were considered, but no evidence supporting these hypotheses was discovered. In the absence of any data to support alternative hypotheses, and consistent with the hypothesis raised in the Connecticut case that contact with cross-contaminated bulk mail accounted for exposure (*7*), we think that this hypothesis is also reasonable for our NYC case. However we found no direct evidence to support or to refute it.

We cannot definitively conclude, however, that contaminated or cross-contaminated mail was the mechanism of exposure in this isolated case. First, the case-patient was not linked to a known contaminated or threat letter, and environmental testing did not provide any evidence of anthrax spores in her home or workplace. Second, although some potentially cross-contaminated mail from the contaminated digital bar code–sorting machine in Trenton was delivered to her workplace and to an address two blocks from her apartment, similar mail was sent to thousands of other locations in NYC, the metropolitan area, and nationwide. Only one other additional inhalational case was identified in the United States after this case (*13*). More cases of cutaneous or inhalational anthrax might be expected if cross-contaminated mail were the mechanism of transmission for our case-patient.

We also found no evidence of inhalational anthrax cases in the areas where the most heavily contaminated postal distributional centers were located in Washington, D.C., and New Jersey. Moreover, the two unexplained cases in NYC and Connecticut occurred several weeks after the last known contaminated letters were postmarked. Why cases that might have been caused by cross-contaminated mail did not occur closer to the time that cross-contamination likely occurred remains unclear. Although our surveillance may have missed a nonhospitalized person ill with anthrax, no other inhalational cases were identified nationwide. Therefore, if cross-contaminated mail was the source for these two last inhalational cases, the risk for illness after exposure to low levels of spores on secondarily contaminated mail is low, given that potentially millions of letters might have had low-level contamination (*15*) based on the positive environmental findings in numerous postal facilities during the 2001 anthrax outbreak.

We decided to close the case-patient’s workplace several hours after her definitive diagnosis. Four factors were involved in this early decision: 1) whether the hospital was the site of recent aerosolized anthrax was unclear, 2) our case had no obvious link to exposure through the mail, 3) environmental testing was facilitated by a closed facility, and 4) closure put the fewest possible people at risk for further exposure. We benefited from decisions made in other states during other anthrax investigations in the preceding weeks. After the final results of the environmental sampling were available, the hospital was reopened on November 6.

Our entire investigation lasted 6 weeks and involved >100 local, state, and federal investigators. The bulk of the environmental sampling was completed within 7 days, although subway sampling began 2 weeks after the case-patient went to the hospital (Table). The case-patient’s hospital workplace was not forced to close, and employees were back at work 10 days after she was diagnosed.

Some limitations exist in our investigation of this case. The case-patient’s rapid death prevented investigators from interviewing her directly to assess potential sources of exposure, including contact with suspicious mail or persons. Given the absence of additional inhalational cases in NYC for comparison, we could only speculate about the activities that represented risk factors for her infection. Unlike the subsequent Connecticut inhalational anthrax case, we could not account for a large proportion of our case-patient’s time when exposure could have occurred. Low-level contamination from mail in her home, workplace, or NYC postal facilities may also have been cleaned up before environmental testing during the 16-day period after these letters were processed. Finally, our environmental sampling strategy was based on the assumption that an aerosol release of *B. anthracis* at any of the locations would have resulted in evidence of environmental contamination detectable by the methods used.

All aspects of the epidemiologic and environmental investigation (e.g., interviewing, collecting samples, interpreting results, developing strategy) occurred through active collaboration with multiple local and federal partners, including public health and law enforcement agencies and the involved healthcare facilities. Our investigation underscores how pre-existing relationships and preplanning among local response agencies fosters the coordination and collaboration needed between the parallel epidemiologic and criminal investigations during an acute crisis (*16*).

This case also emphasizes the important roles that epidemiologic and environmental investigations play in shaping decisions regarding appropriate antibiotic prophylaxis during suspected bioterrorist events. Co-workers, patients, and hospital visitors were initially offered antibiotic prophylaxis when we were uncertain whether an aerosolized release had occurred in her workplace. Given the short incubation period for inhalational disease and the high death rate if treatment is not started early, we decided to initially offer antibiotic prophylaxis to potentially exposed persons. After the initial investigation failed to identify additional suspected cases or any evidence of anthrax spores in the case-patient’s work environment, we recommended discontinuing antibiotics. If any of the environmental samples collected from her workplace had yielded *B. anthracis* isolates, we would have extended the duration of prophylaxis to >60 days to those persons deemed to be at risk for inhalational disease on the basis of the location of the positive results. The liberal use of broad antibiotic prophylaxis earlier in the 2001 anthrax outbreak is thought to have prevented many cases of inhalational anthrax (*17*).

Our investigation provided a number of lessons for future such investigations. We learned that a rapid and adaptive decision-making process regarding scientific issues is necessary and that this process must use both governmental and academic advisors. Laboratory surge capacity is required to continue vital public health activities and respond to a crisis, and surveillance systems for bioterrorism incidents are needed that can be used routinely with familiarity by staff. Health department staff must be familiar with environmental testing and decontamination; proactive information management with early media presence and key officials who are informed is essential.

DOH, in cooperation with multiple local and federal agencies, investigated a single, fatal case of inhalational anthrax whose genotypic similarity to other cases of intentional anthrax in the 2001 anthrax outbreak indicates it was bioterrorism-related. The place and route of exposure to *B. anthracis* in this isolated case remain unknown. Unless the criminal investigation yields further answers, we will likely never know the source of infection or method of exposure in this case. This case highlights the challenges in explaining single bioterrorism-related illnesses with standard epidemiologic and environmental methods and underscores the need for coordination between public health officials and law enforcement agencies during bioterrorism-related investigations.
